# Unmasking the Myositis: A Diagnostic Clue to Occult Breast Cancer

**DOI:** 10.7759/cureus.109270

**Published:** 2026-05-20

**Authors:** Kavuru Naga Siri, Ravi Chandran Ambalathandi, Naveen Ravel, Ramya Lakshmi

**Affiliations:** 1 Medical Oncology, Sri Ramachandra Institute of Higher Education and Research, Chennai, IND

**Keywords:** breast carcinoma, cancer-associated myositis, dermatomyositis, gottron papules, gottron's papules, malignancy, occult, paraneoplastic, physical examination, proximal limb weakness

## Abstract

Dermatomyositis (DM) is an idiopathic inflammatory myopathy characterised by proximal muscle weakness and pathognomonic cutaneous features. A well-recognised but underappreciated association exists between DM and underlying malignancy, termed cancer-associated myositis (CAM). The prevalence of malignancy in DM patients is high, with breast, ovarian, lung, and gastrointestinal cancers most commonly implicated. However, the detection of occult malignancy in young women presenting with DM remains particularly challenging due to atypical epidemiology and the limitations of standard imaging in dense breast tissue. This report presents the case of a woman in her third decade who was initially diagnosed with idiopathic DM based on heliotrope rash, Gottron papules, proximal limb weakness, elevated creatine phosphokinase (CPK), and serological positivity for anti-Mi-2 antibody. Despite the initiation of corticosteroids and methotrexate, the patient showed clinical deterioration over four weeks. Discovery of a firm left axillary lymph node on physical examination prompted further workup. Axillary node biopsy confirmed metastatic estrogen receptor-positive(ER+)/progesterone receptor-positive (PR+)/human epidermal growth factor receptor 2 (HER2)-neu+ breast adenocarcinoma. Conventional mammography and breast ultrasonography had been negative, while breast MRI identified a 1.8 mm lesion in the lower inner quadrant. Fluorodeoxyglucose (FDG)-PET/CT confirmed locoregional disease without distant metastasis. Following neoadjuvant chemotherapy, surgery, and radiation, the patient achieved complete remission of her myositis, with normalisation of creatine phosphokinase (CPK) levels, resolution of rash, and recovery of muscle strength, confirming the paraneoplastic aetiology. This case underscores the imperative to pursue malignancy screening in DM patients unresponsive to immunosuppressive therapy, regardless of age. It highlights the critical role of thorough physical examination and the superiority of breast imaging in young women with dense breasts.

## Introduction

Dermatomyositis (DM) is a systemic autoimmune condition classified among the idiopathic inflammatory myopathies. It is characterised by progressive proximal muscle weakness accompanied by distinctive cutaneous manifestations, including heliotrope rash, Gottron papules, mechanic’s hands, and periungual changes. The annual incidence is estimated at approximately 1-10 cases per million, with a female preponderance and bimodal age distribution, affecting both children and adults [1].

The association between DM and internal malignancy has been firmly established in the literature. Cancer-associated myositis (CAM) is diagnosed when a neoplasm is identified within three years of the DM diagnosis, with malignancy risk elevated three- to seven-fold compared to the general population [2]. The most commonly associated cancers include ovarian, lung, gastric, colorectal, and breast carcinomas. CAM in women younger than 35 years, however, is exceedingly rare and may not be systematically screened for in routine clinical practice.

The diagnosis of occult breast carcinoma in young women presents unique challenges. Dense breast parenchyma, common in premenopausal women, significantly reduces the sensitivity of mammography and conventional ultrasonography, allowing lesions to remain undetected [3,4]. Breast MRI, with its superior soft-tissue resolution, has emerged as an indispensable tool in this population [5].

## Case presentation

**Figure 1:**
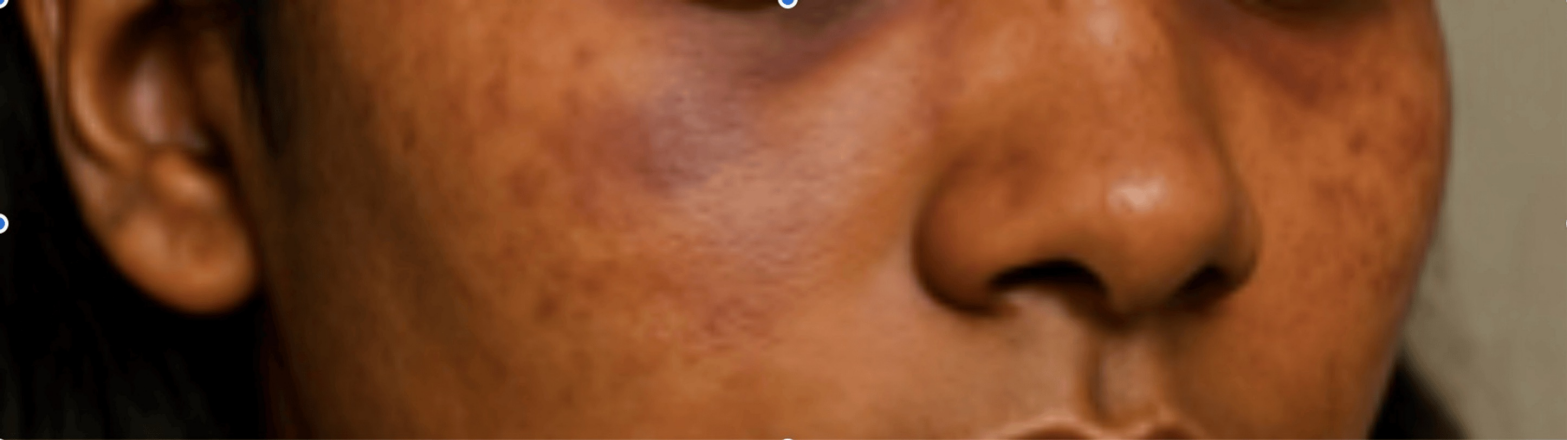
Rash on face

**Figure 2:**
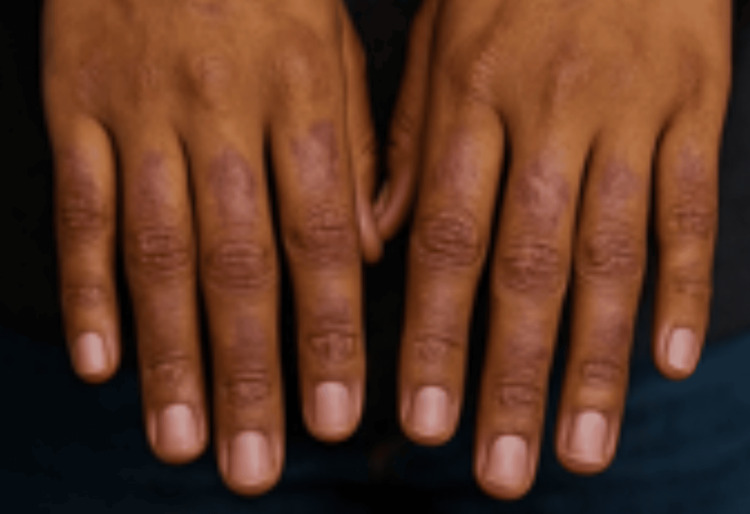
Gottron pappules

**Figure 3:**
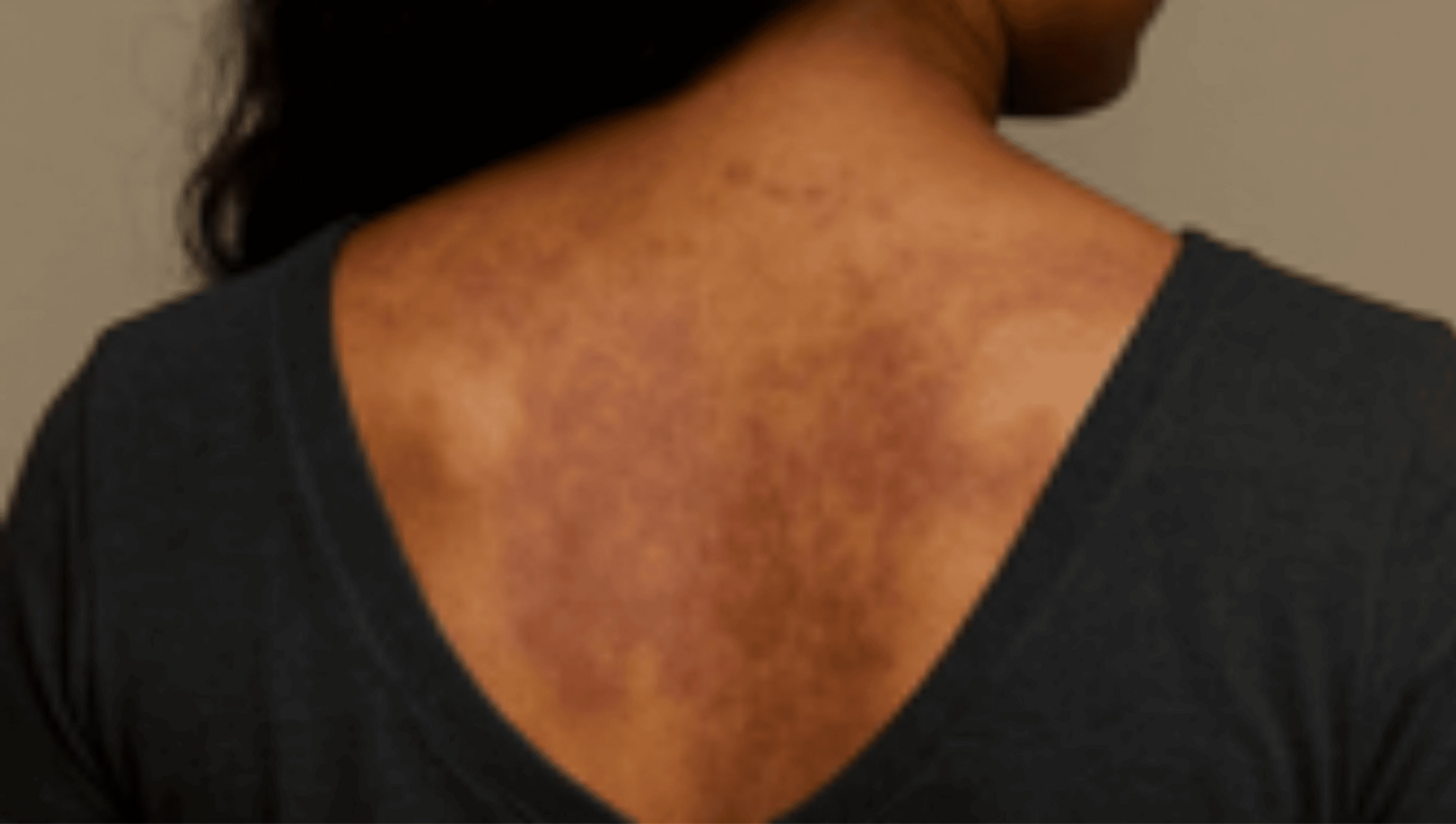
Rashes on trunk

A young woman in her third decade with no significant past medical history presented to the hospital with a six-week history of progressive proximal limb weakness, photosensitivity, and a distinctive facial rash. The classic heliotrope discoloration of the periorbital skin was seen (Figure 1). On examination, erythematous, flat-topped papules overlying the metacarpophalangeal and proximal interphalangeal joints were consistent with Gottron papules (Figure 2). Additional cutaneous involvement, including periungual erythema and poikilodermatous changes on the anterior chest were seen (Figure 3).

Laboratory investigations revealed markedly elevated creatine phosphokinase (CPK) at 3,120 IU/L, a positive antinuclear antibody (ANA) at a titre of 1:160 with a speckled pattern, and serological positivity for anti-Mi-2 antibody. Electromyography (EMG) demonstrated a myopathic pattern with spontaneous activity, and muscle MRI confirmed symmetric oedema in the proximal musculature of both upper and lower limbs. A diagnosis of DM was established in accordance with the Bohan and Peter criteria.

The patient was commenced on oral prednisolone at 1 mg/kg/day in combination with methotrexate 15 mg weekly. Despite four weeks of therapy, she demonstrated no clinical improvement; muscle weakness worsened, and CPK remained elevated. Routine follow-up physical examination revealed a 2×2 cm firm, mobile node in the left axilla. Breast examination was otherwise unremarkable to palpation bilaterally.

Given the steroid-refractory course and the unexpected axillary finding, malignancy workup was expedited. Initial bilateral mammography and breast ultrasonography were reported as negative. Subsequent breast imaging identified a 1.8 mm enhancing lesion in the lower inner quadrant of the left breast, classified as Breast Imaging Reporting and Data System (BI-RADS) 4. Fluorodeoxyglucose (FDG)-PET/CT confirmed locoregional disease confined to the left breast and ipsilateral axillary nodes, with no evidence of distant metastasis. Excisional biopsy of the axillary node confirmed metastatic adenocarcinoma with an estrogen receptor-positive (ER+)/progesterone receptor-positive (PR+)/human epidermal growth factor receptor 2 (HER2)-neu+ receptor profile.

The patient was managed with neoadjuvant chemotherapy comprising a trastuzumab-based regimen, followed by modified radical mastectomy and adjuvant radiation therapy. Immunosuppressive therapy for DM was tapered concurrently. Within eight weeks of initiating oncological treatment, CPK levels normalised, proximal muscle strength recovered to baseline, and the cutaneous manifestations resolved completely. The clinical course confirmed the paraneoplastic aetiology of her DM.

## Discussion

This case presents a compelling illustration of CAM in an atypical demographic, a woman in her third decade, where the clinical index of suspicion for underlying malignancy may be low. The literature consistently identifies older age, male sex, and specific myositis-associated antibody profiles (particularly anti-TIF1-γ and anti-NXP2) as risk factors for CAM, and inflammatory mediators including interleukin (IL)-6 and IL-12 have been implicated in its pathogenesis [1]. However, as this case demonstrates, the absence of conventional risk factors should not preclude a thorough malignancy evaluation when clinical behaviour is atypical [2].

Steroid-refractory DM is a pivotal clinical red flag. A failure to respond to adequate immunosuppression within four to six weeks warrants reassessment of diagnosis and urgent exclusion of underlying neoplasia [1]. In published series, between 15% and 32% of adult DM patients are ultimately found to harbour a concurrent malignancy [2], and a proportion of these present with refractory disease as the initial manifestation. The temporally parallel course of myositis and tumour biology in this patient is consistent with the paraneoplastic hypothesis: shared antigenic epitopes between tumour-associated antigens and regenerating muscle fibres are thought to incite a cross-reactive autoimmune response, perpetuated by tumour progression and abated by effective oncological treatment [6,7].

Breast carcinoma is among the most frequently associated malignancies in female DM patients [2]. HER2-positive tumours, as observed in this case, are of particular interest in the context of paraneoplastic syndromes. HER2 overexpression is associated with heightened immunogenicity, and several reports have documented paraneoplastic neurological and myopathic syndromes in association with HER2-positive breast cancer [6,7]. While anti-Mi-2 positivity is generally associated with a lower malignancy risk compared to anti-TIF1-γ, it does not exclude CAM, and this case reinforces the importance of comprehensive screening irrespective of antibody status [1,6].

The diagnostic challenge in young women with dense breast tissue is well-documented. In this case, standard mammography and ultrasonography were entirely negative despite active locoregional disease; it was breast MRI with contrast-enhanced dynamic sequences that enabled the detection of a sub-centimetre lesion that would otherwise have remained occult, demonstrating superiority over digital breast tomosynthesis in women with dense parenchyma [3]. This limitation of conventional imaging is well characterised: mammographic sensitivity is reported to decrease from approximately 87% in fatty breasts to as low as 62% in extremely dense breasts [4], whereas MRI sensitivity approaches 90% even in dense breast tissue [5]. The American College of Radiology now recommends supplemental MRI screening for women at intermediate-to-high lifetime risk, and cases such as this support broader application of this modality when clinical suspicion warrants investigation beyond conventional imaging [8].

The role of meticulous physical examination deserves emphasis. In this patient, it was routine axillary palpation, not advanced imaging, that revealed the critical finding that prompted oncological workup. In resource-limited settings where advanced imaging is not always immediately accessible, clinical examination remains an irreplaceable diagnostic tool. Comparable cases in the literature have similarly identified axillary lymphadenopathy as the presenting feature of occult breast malignancy in DM patients [6,7,9]. The complete remission of DM following oncological treatment is the most compelling evidence of its paraneoplastic aetiology. This pattern has been replicated across multiple published case series, wherein resolution of myositis tracks closely with tumour response, and relapse of myositis may herald disease recurrence [7,9]. The therapeutic implication is clear: in CAM, definitive treatment of the primary malignancy is the cornerstone of myositis management, and immunosuppressive agents serve only as adjunctive, temporising measures. The need for further research on autoantigens implicated in this cross-reactive immune response remains of great importance [10].

## Conclusions

This case affirms that DM may serve as a paraneoplastic herald of occult breast carcinoma, even in young women without conventional risk factors for malignancy. Steroid-refractory DM must prompt urgent and systematic oncological workup, inclusive of breast MRI when standard imaging is unrevealing. Comprehensive physical examination, including axillary assessment, remains indispensable. Resolution of myositis following cancer-directed therapy confirms the paraneoplastic diagnosis and underscores the primacy of treating the underlying malignancy.
